# Mydriatics

**Published:** 1891-11-14

**Authors:** 


### OPHTHALMOLOGY.

### MYDRIATICS.

Mydriatics are used:—

(1) To dilate the pupil (*a*) for ophthalmoscopic examination; (*b*) in iritis to prevent adhesion of the iris to the posterior capsule of the lens, or the deposit of uveal pigment on the central portion of the cornea, where it would interfere with clear vision, by obstructing the rays of light as they entered the eye. (*c*) In cases of corneal wound, either during operation, ulceration, or from a traumatic source, to draw the iris out of reach of the wound, and thus prevent its prolapse through the wound. (*d*) In incipient cataract to admit more light, especially where the central portion of the lens is affected.

(2) To act mechanically, where posterior synechiæ exist, by dragging the iris from the lens capsule, the circular muscular fibres being rendered inert, the radial fibres by virtue of their tonic dilating influence tend to drag away from the centre, and thus break down the adhesions of the iris to the more convex part of the lens capsule.

(3) To paralyse accommodation (*a*) for estimating the refraction of the eye. (*b*) In cases of ciliary spasm from overstrain of the muscle. (*c*) In internal strabismus in young children, to prevent them using the near vision, which favours convergence.

How do mydriatics act? Is it by paralysing the third nerve which governs the radial muscular fibres, or contracting muscular fibres of the iris; or by stimulating the sympathetic nerves which govern the radial or dilating muscular fibres of the iris?

To arrive at a conclusion we must remember that stimulation of the retina by light acts through a reflex mechanism, in which the second or optic nerve is the afferent nerve, and the third the efferent nerve, causing contraction of the pupil, and this stimulus being removed the tonic nature of the radial fibres causes dilatation of the pupil, the sympathetic being the efferent nerve. If, therefore, the third nerve, the efferent nerve for contraction, be divided, the pupil will dilate, the stimulus of light being checked from passing through this nerve to the muscles of contraction, and the dilating mechanism will have full play; in fact, the third nerve is paralysed. If, therefore, the mydriatic acts by paralysing the third nerve, the application of atropine should have no effect on a dilated pupil after the division of the aforesaid nerve, but as a matter of fact the application of the drug still further increases the dilatation, showing that it must affect some other mechanism. Again, the ophthalmic ganglion's agency is excluded in the same way, as after its excision dilatation will be still further increased by exhibiting atropine. It must, therefore, act on a local mechanism, ganglionic nerve-cells locally situated in the ciliary region.

Mydriatics act both by internal administration and local application; the latter is the mode usually employed, but in an article on the subject we must refer to both, and classify them thus:—

(1) By internal administration or hypodermic injection—Atropia, belladonna, daturine, duboisine, homatropine, hyoscyamine, nicotine, and by inhalation chloroform, in later stages.

(2) By local application—Atropia, belladonna, daturine, duboisine, homatropine, hyoscyamine, cocain, gelsemia, muscarine. Gelsemia and muscarine, when administered internally, cause contraction of the pupil.

The local application of these drugs may be in the form of solutions, ointments, or discs made by combination with gelatine, each mode having its own advocates. The application should be made carefully and scientifically, with a view to obtaining the best local effect, without introduction into the general system. In using the solutions the operator should lift the upper eyelid with the forefinger of the left hand, and draw away the punctum lachrymalis with his thumb. Then directing the patient to look downwards, drop one drop of the solution at a time on the surface of the eyeball at its outer and upper part. Absorption will then take place through the lymph channels of the cornea and conjunctiva, and not pass into the nasal duct, and thus into the general circulation. The ointment and discs are best applied at the same part; the ointment spreads over the eyeball, and being of a greasy nature, does not mix readily with the tears, which, in the case of the solutions, carry the drug into the nasal duct, unless the punctum lachrymalis is held well away from the eyeball. The discs dissolve comparatively slowly, and by them, whether the absorption be wholly through the ocular tissues or the nasal membrane, the dose can be exactly regulated, each disc containing a definite amount of the drug employed.

In ophthalmic practice mydriatics are almost universally used locally, and *Atropine* is by far the most frequently used drug, rapid and full dilatation is easily obtained with a 1 per cent. solution, the Liq. Atropinæ Sulphatis of the B.P. 1885. The above diluted with an equal quantity of distilled water is a very useful strength to use, a single drop of such solution applied to the eye causes dilatation of the pupil to commence in a quarter of an hour, full dilatation taking about from half an hour to three-quarters of an hour, and complete paralysis of accommodation about two hours; the full effect lasts twenty-four hours, and the recovery takes from three days to a week, the stronger the solution the longer the effect lasts. In cases of ciliary spasm this solution, or ointment or discs of corresponding strength, require to be used thrice daily. This may be kept up for a week or ten days, and it will take from eight to ten days to recover from it, the accommodative power returning before the pupil has quite recovered. When in incipient cataract the pupil is to be dilated to admit more light a much weaker solution from ·1 per cent. to ·05 per cent., applied every day or every second day will suffice.

Sometimes Atropine produces an inflammation of the conjunctiva, in such cases one of the other mydriatics must be substituted. *Sulphate of Daturine*, 1 p.c. or *Sulphate of Duboisine*, ·25 p.c., are often tolerated where Atropine Sulphate is not, and act efficiently, their action is quicker, and less prolonged, dilatation commencing in ten minutes, full dilatation is completed in twenty minutes, and paralysis of accommodation in an hour. Duboisine must be used carefully as it often produces symptoms of poisoning. Duboisine and Daturine Sulphates are 8d. a grain. *Homatropine* is particularly useful, as full dilatation can be obtained with a 1 p.c. solution in about half-an-hour, and paralysis of accommodation in one hour, the patient recovering from the effects within the twenty-four hours, which is often the limit of time a person can afford to do without accommodation. The Hydrobromate of Homatropine is the salt usually employed. Its only drawback is its price, 1s. 3d. a grain. *Cocain Hydrochloratis* 4 p.c. is a useful mydriatic where temporary dilatation for ophthalmoscopic examination is needed; it takes somewhat longer to produce dilatation with it, than with the afore-mentioned; but the recovery from its effects is much more rapid, seldom exceeding four hours, even after frequent applications. *Hyoscyamine* acts like atropine, but with greater intensity: the duration of the effect is also about the same; it is not much used for its mydriatic properties. *Gelsemina Hydrochloratis* is a rapid mydriatic when applied locally: maximum dilatation occurs in about one hour, and the effect passes off somewhat quicker than does that of atropine. A 2 p.c. solution may be used. It costs about 9d. a grain. *Muscarine*, the alkaloid of a fungus, is also a mydriatic when applied locally, but is not used in practice. With such a large choice, the practitioner must take into account the various *pros* and *cons* for using one or another, the effect required, the time it is necessary to paralyse accommodation for, the tendency of the drugs to produce toxic symptoms if not carefully applied, the price of the drugs in the market, &c.

By hypodermic injection the various mydriatics act rapidly, but produce effects on the whole system, *pari passu*. By internal administration the system becomes affected also, the pupil dilating as the general effect is produced. The ophthalmic surgeon, therefore, always uses the mydriatics locally.

